# Prevalence and genetic diversity of *optrA*-positive enterococci isolated from patients in an anorectal surgery ward of a Chinese hospital

**DOI:** 10.3389/fmicb.2024.1481162

**Published:** 2024-11-08

**Authors:** Yuanyuan Li, Tao Jiang, Jianfeng Mao, Fangyi Xu, Rong Zhang, Jing Yan, Jiachang Cai, Yanjun Xie

**Affiliations:** ^1^Department of Clinical Laboratory, The First Affiliated Hospital of Henan University of Science and Technology, Luoyang, China; ^2^Department of Clinical Laboratory, Affiliated Xiaoshan Hospital, Hangzhou Normal University, Hangzhou, China; ^3^Department of Clinical Laboratory, Second Affiliated Hospital of Zhejiang University, Hangzhou, China

**Keywords:** *optrA*, linezolid resistance, *enterococci*, genetic context, hospital

## Abstract

Linezolid-resistant enterococci have increased in recent years due to the worldwide spread of acquired resistance genes (*cfr*, *optrA*, and *poxtA*) in clinical, animal, and environmental settings. This study investigated the carriage of *optrA*-positive enterococci among patients in the anorectal surgery ward in Hangzhou, China, and characterized the genetic context of *optrA*. A total of 173 wound secretion samples were obtained to screen *optrA*-positive enterococci. Of the 173 samples, 15 (8.67%) were positive for *optrA*, including 12 *Enterococcus faecalis*, two *E. faecium*, and one *E. hirae*. Multilocus sequence type analysis revealed that 12 *optrA*-positive *E. faecalis* isolates belonged to eight different sequence types (STs), of which ST16 was the main type. Eight optrA variants were identified, whose *optrA* flanking regions with a *fexA* gene downstream were bounded by different mobile genetic elements. Furthermore, the *optrA* gene in 8 out of 15 *optrA*-positive enterococci could be successfully transferred through conjugation. The findings revealed a high carriage rate of *optrA* in enterococci from one anorectal surgery ward in China. The dissemination of *optrA*-positive enterococci isolates in clinical settings should be continually monitored.

## 1 Introduction

Enterococci are important commensal bacteria found in the intestines of humans and many animals. However, they can also cause hospital-acquired infections, including meningitis, bacteremia, pneumonia, surgical wound infections, and urinary tract infections (Yi et al., 2022). The emergence of multidrug-resistant strains has complicated the treatment of enterococcal infections.

Linezolid is an oxazolidinone antimicrobial agent that is exclusively used for the treatment of severe infections caused by vancomycin-resistant enterococci (VRE), methicillin-resistant *Staphylococcus aureus* (MRSA), and *penicillin-resistant pneumococci* (Echeverria-Esnal et al., 2019). However, the extensive use of linezolid results in the emergence of resistance. The main mechanism mediating resistance to linezolid has been attributed to mutations in the central loop of the domain V region of the 23 s rRNA gene. In addition, acquired resistance genes (*cfr*, *poxtA*, and *optrA*) were identified (Zhang et al., 2023; Partridge et al., 2018). Since its first description in 2015, *optrA* has been frequently reported in enterococci of human, animal, and environmental origins from many countries (Andrea et al., 2015; Mendes et al., 2016; Flamm et al., 2016; Gawryszewska et al., 2017; Na et al., 2020; Han et al., 2020; Li et al., 2020). Furthermore, *optrA* has been reported in Gram-positive bacteria including *Enterococcus*, *Staphylococcus*, and *Streptococcus* as well as Gram-negative bacteria such as *Campylobacter* and *Salmonella* (Zhang et al., 2023; Liu et al., 2020; Schwarz et al., 2021). The *optrA* gene is often located on chromosomes or plasmids and can be transmitted by mobile genetic elements such as transposons and insertion sequences (Chen et al., 2018).

Enterococci can readily acquire or transfer multidrug resistance genes via mobile genetic elements and are the most predominant source of the spread of *optrA*. Enterococci harboring *optrA* have been widely detected in clinical, farm, and environmental settings worldwide (Gagetti et al., 2023). The dissemination of *optrA* is a serious concern and poses a potential public health threat. The prevalence of *optrA*-positive *E. faecalis* was 0.2% in Austria (Kerschner et al., 2021) and 0.7% in Spain (Rodríguez-Lucas et al., 2022). A 3-year survey in Korea showed that 0.23% of clinical *E. faecalis* isolates harbored the *optrA* gene (Park et al., 2020). However, in China, the positive rate of *optrA* has increased from 0.4% in 2004 to 3.9% in 2014 (Cui et al., 2016). Therefore, the prevalence and spread of *optrA*-carrying enterococci should be monitored carefully. The *optrA*-positive *E. faecalis* emerged in a tertiary care hospital (Park et al., 2020). Several studies have reported fecal carriage rates of 3.53% for *optrA*-positive enterococci in healthy individuals and 15.1% in patients who underwent anorectal surgery (Cai et al., 2019; Cai et al., 2016). *optrA*-positive enterococci may cause transmission of resistance genes in the intestine. However, reports of *optrA*-positive enterococci from anorectal infections have been limited. In this study, we investigated the prevalence of the *optrA*-positive enterococci isolates from an anorectal surgery ward in a Chinese hospital. We utilized whole-genome sequencing (WGS) to further describe the *optrA* genetic context.

## 2 Materials and methods

### 2.1 Sample collection and bacterial isolation

A total of 173 non-duplicated wound secretion samples were collected from an anorectal surgery ward (118 male and 55 female patients) in a tertiary care hospital in Hangzhou, China. Each sample was collected from a different patient: 108 samples were taken from patients with perianal abscess and 65 samples were taken from patients with appendicitis. All the samples were processed to screen for isolates harboring *optrA* as we previously described (Shen et al., 2022). Briefly, 20 mg of each wound secretion sample was inoculated into 5 ml of Luria-Bertani (LB) broth (Beijing AOBOX Biotechnology, Beijing, China) within 4 h of collection and incubated at 37°C for 24 h. A volume of 100 ml from each enriched sample was transferred to 5 ml fresh LB broth containing 5% NaCl and 10 mg/L florfenicol (Shanghai Aladdin Biochemical Technology, Shanghai, China) and subcultured for 24 h. Then, 20 ul of each resulting culture was streaked onto a selective medium consisting of Columbia agar (Autibio, Henan, China) base supplemented with 5% (v/v) sheep blood (Biolife Italiana S.r.l., Milan, Italy) and 10 mg/L florfenicol and incubated at 37°C for 24 h.

### 2.2 Species identification and detection of oxazolidinone-resistance genes

Based on the colony morphology, putative target isolates were selected from the developed colonies. Species identification was performed using matrix-assisted laser desorption ionization–time of flight mass spectrometry (Bruker Daltonik GmbH, Bremen, Germany). All florfenicol-resistant enterococci isolates were again subcultured for purification and then screened for the presence of *optrA*, *poxtA*, *cfr*, and *cfrD* genes using PCR and Sanger sequencing, following previously described procedures (Li et al., 2020). The following primers were used (Shang et al., 2019; Antonelli et al., 2018; Kehrenberg and Schwarz, 2006; Coccitto et al., 2023; *optrA*-F: GCACCAGACCAATACGATACAA, *optrA*-R: TCCTTCTTAACCTTCTCCTTCTCA, *poxtA-*F: GGTCTGACTGGCTTGTTTTGCT, *poxtA-*R: ATAAGGTCGGTATTGTCGGCGT, *cfr-*F: TAAGAAGTAATAATGAGC, *cfr-*R: TATAGAAAGTCTACGAGG, *cfr(D)-*F: TGCGCTACTGGAAAAATTGGC, and *cfr(D)-*R: GCTTGAACGTTCTTGGTGCAT).

### 2.3 Antimicrobial susceptibility testing

The minimum inhibitory concentrations (MICs) of seven antimicrobial agents (Shanghai Aladdin Biochemical Technology, Shanghai, China) were determined using the broth microdilution method (CLSI, 2018; Tang et al., 2022). The antimicrobial agents were linezolid, chloramphenicol, penicillin G, vancomycin, ciprofloxacin, erythromycin, and tetracycline. Broth microdilution was performed in Brucella broth supplemented with 2% fetal calf serum. Twofold dilutions of each antimicrobial agent ranging from 0.125 to 256 μg/ml were used. To each plate, 100 μl was added with an inoculum concentration of approximately 5 × 10^7^ colony-forming units (CFUs)/ml. The plates were incubated for 24–48 h at 37°C. The MIC was defined as the lowest concentration of the drug. The results were interpreted according to the Clinical and Laboratory Standards Institute standard (CLSI M100-Ed32; CLSI, 2022).

### 2.4 Conjugation experiment

To investigate the transferability of *optrA*, *poxtA*, and *cfrD*, conjugation experiments were performed using the filter-mating method with rifampicin-resistant *E. faecalis* JH2-2 as a recipient (Hao et al., 2019; Xie et al., 2021). Briefly, the donor and recipient were cultured in fresh LB broth at 37°C for 4 h to reach the logarithmic phase. Then, the donor and recipient were mixed at a ratio of 1:4 and then incubated on a 0.45-μm membrane placed on an LB agar plate for 24 h at 37°C. Transconjugants were selected on LB agar (TSA) plates supplemented with 30 mg/L rifampicin and 10 mg/L florfenicol. Colonies that grew on these selective plates were chosen after incubation for 16–24 h at 37°C. The presence of *optrA*, *poxtA*, and *cfrD* and species identification of the transconjugants were confirmed using PCR and MALDI TOF/MS, respectively. Conjugation frequency was determined as the number of transconjugants/the number of recipients.

### 2.5 Whole-genome sequencing and genome analysis

Total genomic DNA was extracted from overnight cultures of 15 isolates using the PureLink Genomic DNA Mini Kit (Invitrogen, Carlsbad, CA, USA) according to the provided instructions. WGS was performed using the NovaSeq 6,000 platform (Illumina, San Diego, CA, USA). The sequencing data were *de novo* assembled into contigs by SPAdes v.3.13.1 (Hölzer and Marz, 2019). Antimicrobial resistance genes were analyzed using the ResFinder2.1 bioinformatic database.^1^ Plasmid replicons were identified using PlasmidFinder (2.1).^2^ A heatmap of antimicrobial resistance genes was performed using Morpheus.^3^ Multilocus sequence typing (MLST) of strains was performed using the PubMLST tool.^4^ Plasmid sequences were initially annotated^5^ using a subsystem technology (RAST version 2.0) server and curated manually using the BLASTn and BLASTp algorithms.^6^ Easyfig (v2.2.2) was used to visualize the linear alignment of the genetic environment of the *optrA* gene in different isolates.^7^

### 2.6 Phylogenetic analysis using core-genome single-nucleotide polymorphism

Trimmed and quality-filtered assembly sequences of 12 *optrA*-*E. faecalis* were aligned with the reference strain GZ86, and the phylogenetic trees of the isolates were constructed using Parsnp v2.0.3 based on core genomic single-nucleotide polymorphism (cgSNPs).^8^ The phylogenetic tree was visualized and retouched using iTOL.^9^

## 3 Results

### 3.1 Characteristics of enterococci harboring *optrA*

In this study, 15 florfenicol-resistant enterococci were obtained from 173 wound discharge samples. All samples were positive for *optrA* with a carriage rate of 8.67% (15/173). Among the *optrA*-carrying isolates, *E. faecalis* had the highest frequency (80%, 12/15), while two *E. faecium* and one *E. hirae* were also isolated.

The carriage rates of *optrA* for male patients and female patients were 8.47% (10/118) and 9.09% (5/55), respectively. The median age of the patients was 32 (IQR: 26–47) years (Table 1). Among the differentially diagnosed diseases, the *optrA* carriage rates varied. In total, 7.4% (8/108) of the patients had perianal abscess and 10.77% (7/65) of the patients had appendicitis. Cephalosporins were used during the treatment period. All patients were discharged.

**Table 1:** Antimicrobial susceptibility results (μg/ml), variants, and clinical information of 15 *optrA*-positive enterococci isolates.

Strain	Enterococci species	variants	Source			Antimicrobial agent MIC(μg/ml)								Conjugation frequency		
			Age	Sex	Clinical diagnosis	VA	TE	LZD	C	P	CIP	E	FF	*optrA*	*poxtA*	*cfrD*
LW22	*E. hirae*	KLDP	32	male	appendicitis	0.5	64	8	32	1	2	8	128	NA	/	/
GZ27	*E. faecalis*	WT	27	male	perianal abscess	0.5	32	8	32	8	8	64	128	5.8 × 10^−6^	/	/
GZ61	*E. faecalis*	RDK	28	male	perianal abscess	0.5	32	8	64	4	8	64	32	7.3 × 10^−5^	4.22 × 10^−5^	4.95 × 10^−5^
GZ83	*E. faecalis*	RDK	25	male	perianal abscess	0.5	32	8	64	8	1	64	64	NA	/	/
GZ86	*E. faecalis*	RDK	50	female	perianal abscess	0.5	64	8	32	4	8	64	128	NA	/	/
GZ133	*E. faecalis*	WT	47	male	perianal abscess	0.5	32	8	64	8	1	64	32	3.23 × 10^−4^	/	/
GZ138	*E. faecalis*	DP	30	female	perianal abscess	0.5	32	8	32	4	8	64	64	1.13 × 10^−3^	/	/
GZ142	*E. faecalis*	DP	21	female	perianal abscess	0.5	32	8	128	4	8	64	32	2 × 10^−3^	/	/
LW158	*E. faecalis*	DP	32	male	appendicitis	0.5	32	8	32	8	8	64	64	NA	/	/
LW161	*E. faecium*	DD	21	male	appendicitis	0.5	32	8	32	32	8	64	64	2.89 × 10^−5^	4.03 × 10^−6^	/
GZ178	*E. faecalis*	DP	47	male	perianal abscess	0.5	32	8	32	8	1	64	32	5.47 × 10^−7^	/	/
LW192	*E. faecalis*	KD	34	male	appendicitis	0.5	32	8	32	4	8	64	32	NA	/	/
LW226	*E. faecalis*	EYDNDM	47	female	appendicitis	0.5	32	4	32	16	8	64	32	NA	/	/
LW227	*E. faecium*	EDM	26	female	appendicitis	0.5	32	4	64	64	2	64	64	NA	/	/
LW233	*E. faecalis*	RDK	33	male	appendicitis	0.5	32	8	32	8	1	64	64	1.71 × 10^−3^	/	/

*VA, Vancomycin; TE, tetracycline; CIP, ciprofloxacin; E, erythromycin; C, chloramphenicol; P, penicillin; LZD, linezolid; FF, florfenicol; NA, not available.*

### 3.2 Antimicrobial susceptibility and identification of *optrA* variants

All *optrA*-positive enterococcal strains were resistant to erythromycin and chloramphenicol (Table 1). All the isolates were either intermediate or resistant to linezolid and exhibited MICs of 4 or 8 μg/ml. Two *E. faecium* isolates were resistant to penicillin, as was one *E. faecalis* isolate with an MIC of 16 μg/ml. The isolate *E. faecalis* GZ27 was susceptible to tetracycline with an MIC of 1ug/ml. No vancomycin-resistant enterococcal strains were isolated.

A total of 8 different optrA variants (including the WT) were identified among the 15 *optrA*-positive enterococci (Table 1). The RDK and DP variants, were the common variants (*n* = 4), followed by WT (*n* = 2). The RDK and DP (*n* = 4) variants were common among *E. faecalis*. One each of the DD, EDM, and KLDP variants was detected in *E. faecium* and *E.hirae*. Among the seven optrA variants, two variants only (EDM and EYDNDM) showed intermediate resistance to linezolid (MIC = 4 μg/ml), whereas the remaining variants (RDK, DP, DD, EDM, and KLDP) showed resistance to linezolid (MIC = 8 μg/ml).

### 3.3 Transferability of *optrA*, *poxtA*, and *cfrD*

To investigate the transferability of *optrA*, *poxtA,* and *cfrD*, all 15 *optrA*-positive isolates were subjected to conjugation experiments. The *optrA* gene in eight isolates could be successfully transferred to *E. faecalis* JH2-2 but could not be in the remaining seven isolates. Conjugation efficiency in the transconjugants of strains differed substantially, ranging from 10^−3^ to 10^−7^ (Table 1). For *poxtA*, the conjugation efficiency in the transconjugants of strains GZ61 and LW161 was 4.22 × 10^−5^ and 4.03 × 10^−6^, respectively. For *cfrD*, the conjugation efficiency in transconjugants of strain GZ61 was 4.95 × 10^−5^.

### 3.4 Genotyping and phylogenetic analysis of *optrA*-positive enterococcal isolates

**Figure 1:**
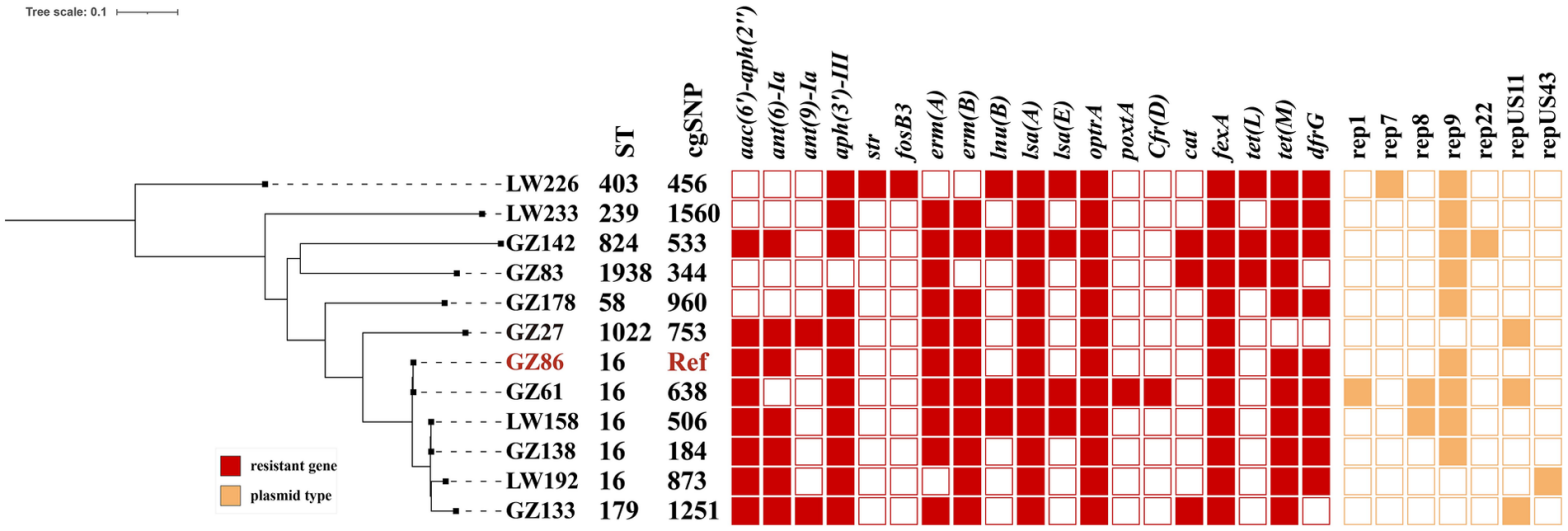
Phylogenetic tree analysis and heatmap of 12 *optrA*-positive *E. faecalis* strains A core-genome Journal Pre-proof phylogenetic tree. Antimicrobial resistance genes, ST, and plasmid types in *optrA*-positive strains are labeled. Colored cells in each column denote the presence of a particular resistance gene or plasmid replicon as labeled at the top. Antimicrobial resistance genes and plasmid replicon types are shown in red and orange, respectively.

MLST analysis revealed 8 different sequence types (STs) among the 12 *optrA-positive E. faecalis* isolates (Figure 1), including ST16 (*n* = 5), ST1022 (n = 1), ST179 (*n* = 1), ST824 (*n* = 1), ST58 (*n* = 1), ST403 (*n* = 1), ST1938 (*n* = 1), and ST239 (*n* = 1). One *E. faecium* isolate belonged to ST885, and another was divided into ST1818 and 244 according to two different parting systems. In this study, it was assigned to ST1818. Nevertheless, phylogenetic analysis based on the SNPs showed that the distribution of *E. faecalis* in an anorectal surgery ward was highly diverse.

WGS revealed multiple antimicrobial resistance genes and plasmid replicons in 12 *optrA*-positive *E. faecalis* strains (Figure 1) and 3 non-*E. faecalis* isolates (Table 2). Each strain was found to carry resistance genes (7–17) and a diverse range of plasmid replicons (1–4). These included aminoglycoside-inactivating enzyme genes [*aac(6′)-Iid*, *aac(6′)-aph(2″)*, *aadD*, *ant(6)-Ia*, *ant(9)-Ia*, *aph(3′)-III*, and *str*], macrolide resistance genes (*ermA*, *ermB*, and *msrC*), phenicol resistance genes (*cfrD*, *cat*, *fexA*, and *fexB*), tetracycline resistance genes [*tet(L)* and *tet(M)*], trimethoprim resistance gene (*dfrG*), oxazolidinone resistance genes (*optrA* and *poxtA*), and fosfomycin resistance gene (*fosB3*). The *fosB3* gene was detected in only one *E. faecalis* LW226. Concerning the phenicol resistance genes, *fexA* was present in all *optrA*-positive strains. Genes less frequently present included *fexB* and *poxtA* (*n* = 2), *cat* (*n* = 3), and *cfrD* (*n* = 1).In addition, other resistance genes including *aac(6′)-aph(2″)* and *dfrG* (*n* = 10), *aph(3′)-III* and *ermA* (*n* = 13), *ermB* and *lsaA* (*n* = 12), and *tet(M)* (*n* = 14) were detected.

**Table 2:** Resistance genes and plasmid replicon types of *optrA*-positive non-*E. faecalis* enterococcal isolates.

Strain species	Resistance gene number	Resistance genes	Plasmid replicons
*E. hirae* LW22	7	*aac(6′)-Iid, erm(A), lun(G), optrA, fexA, tet(L),* and *tet(M)*	repUS1
*E. faecium* LW161	17	*aac(6′)-Iid, aac(6′)-aph(2″), aadD,* and *ant(6)-Ia,* *aph(3′)-III,erm(A), erm(B), ant(9)-Ia, lnu(B), lsa(E), msr(C),optrA, poxtA, fexA, fexB, tet(L),* and *tet(M)*	rep18, rep22, and repUS15
*E. faecium* LW227	13	*aac(6′)-Ii,aac(6′)-aph(2″),aph(3′)-III, fexB, erm(A), erm(B), tet(L), lnu(B), lsa(E), tet(M), optrA, fexA,* and *dfrG*	rep1, rep2, and repUS15

### 3.5 Genomic context of *optrA*

**Figure 2:**
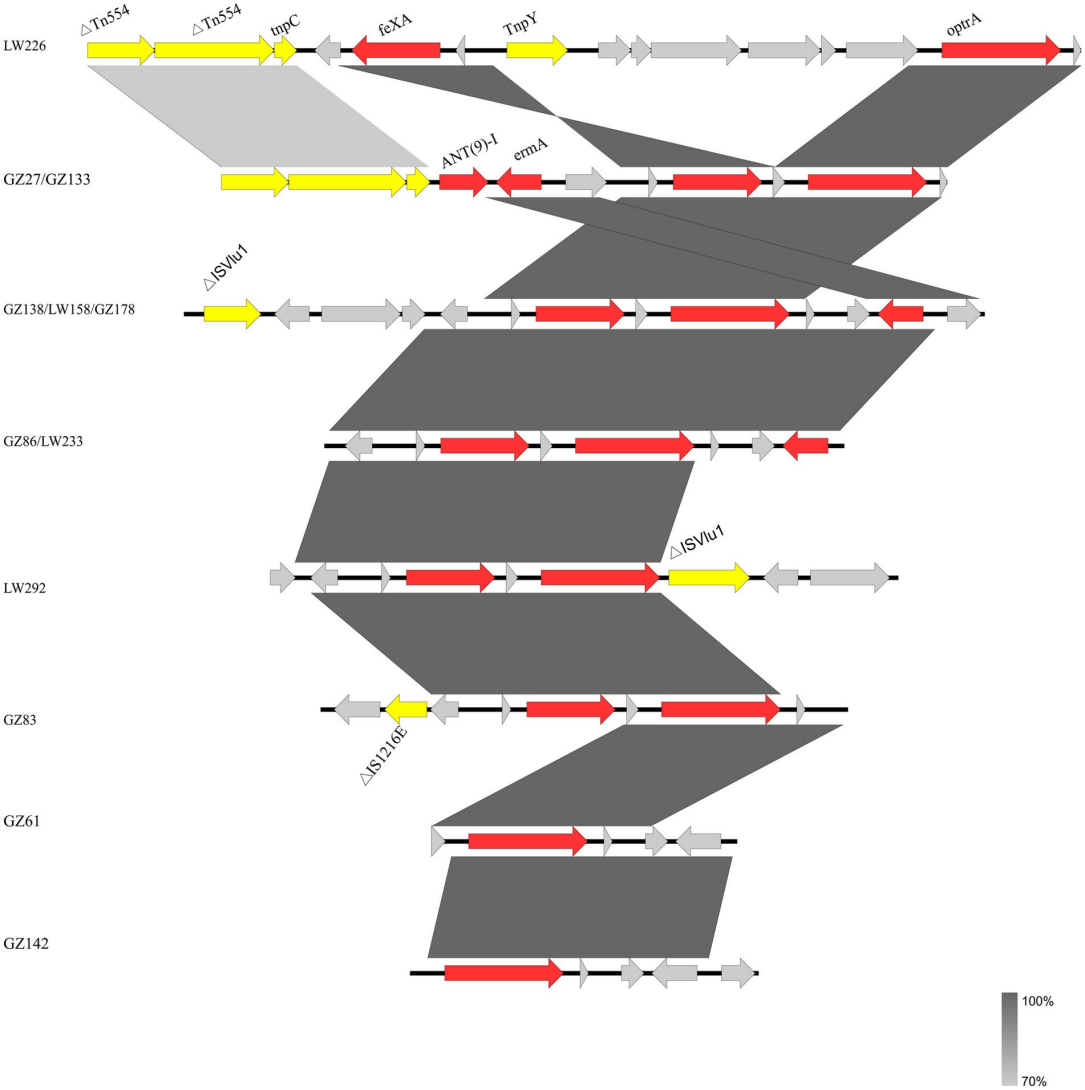
Genetic environment of 12 *optrA*-positive *E. faecalis* isolates in this study. The genes of different functions are labeled with different colors. Δ indicates a truncated gene.

For the 12 *E. faecalis* isolates, identical genetic structures were found in two and three isolates, respectively (Figure 2). The two isolates (GZ61 and GZ142) carried the shortest contig in which only *optrA* was identified.

Several mobile genetic elements including IS1216E, ISVlu1, and transposase genes *tnpC* and *tnpY*, were inserted into the flank structure of *fexA*-*optrA*. Insertion sequences ISVlu1 and IS1216E, belonging to family member ISL3, were detected upstream and/or downstream of the *fexA*-*optrA* fragment. The IS1216E element was located upstream of the *fexA*-*optrA* segment in *E. faecalis* GZ83. The genetic segment *fexA*-*optrA*-ISVlu1 was identified in isolate LW192. Three isolates (GZ138, GZ178, and LW158) shared the same genetic segments ISVlu1-*fexA*-*optrA*-*erm(A)*. Truncated transposon *Tn554* and transposase genes *tnpC* and *tnpY* were located upstream of the *fexA*-*optrA* segments in *E. faecalis* isolates LW226, GZ27, and GZ133. In addition, for the two same genetic environment isolates (GZ27 and GZ133), the resistance genes *ant9-1* and *erm(A)* were present in the region between transposon *Tn554* and *fexA*-*optrA* fragments.

**Figure 3:**
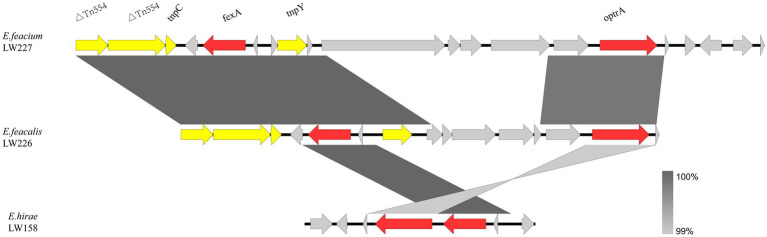
Genetic environment of *optrA*-positive non-*E. faecalis* enterococcal isolates.

The *optrA* flanking regions in three non-*E. faecalis* enterococci are shown in Figure 3. Two *E. faecium* LW227 and LW161 isolates shared the genetic environment *Tn544*-*tnpC*-*fexA*-*tnpY*-*optrA*. No mobile genetic elements were identified in the *E. hirae* strain.

## 4 Discussion

The prevalence of linezolid resistance has rapidly increased. The spread of *optrA*-mediated linezolid resistance in *Enterococcus* could be an imminent threat.

In this study, the prevalence of *optrA*-positive enterococci in wound secretion samples obtained from patients in an anorectal surgery ward of a Chinese hospital was 8.65%. It was 1.1% at a tertiary care hospital in Nanjing and Beijing, China (Zhou et al., 2019; Wang et al., 2024). Several studies showed that fecal carriage rates of *optrA* from healthy humans varied from 3.53% in 2019 to 5.89% in 2022 (Cai et al., 2019; Shen et al., 2022). A previous study reported a human intestinal carriage rate of *optrA* at 19.3%, utilizing an optimized high-sensitivity screening approach (Shen et al., 2022). We used this method to screen for samples. As a result, we discovered an unexpectedly high prevalence. Nevertheless, a study suggested that the prevalence of *optrA* is higher in enterococci from animals than those from humans (Wang et al., 2015; Shen et al., 2024). The isolates from shared bicycles were identified for the presence of *optrA* gene (Han et al., 2020). The *optrA* gene has been widespread in animal, environmental, and clinical isolates, indicating that the horizontal transfer of *optrA* plays a crucial role in the human–animal–environment interfaces (Shen et al., 2024; Gouliouris et al., 2018). In the present study, the patients had no history of linezolid use during hospitalization, indicating that infections were not associated with linezolid use.

In this study, STs and SNPs of *optrA*-positive *E. faecalis* isolated were genetically highly diverse. STs belonged to eight different types, mostly for ST16. ST16, with the GCTGAACC SNP profile, has often been identified in humans, animals, and surface water in various countries (Zhou et al., 2019; Freitas et al., 2020; Bender et al., 2018; Rathnayake et al., 2011). Previous studies reported ST480 as one of the predominant types in France and Germany (Egan et al., 2020). These findings demonstrated the non-clonal dissemination and the widespread presence of *optrA*-positive *E. faecalis* in hospitals.

All strains harbored multiple resistance genes and showed a multidrug-resistant phenotype, which indicated a broad antibiotic resistance spectrum of enterococcal isolates. To our surprise, the fosfomycin resistance gene *foB3* was detected in a single *optrA*-carrying *E. faecalis*. In 2021, the coexistence of *foB3* and *optrA* was the first reported in *E. faecalis* from pigs (Wang et al., 2021). Given that fosfomycin and linezolid are the last-resort antibiotics for treating infections caused by VRE, the co-occurrence of *fosB* and *optrA* in clinical strains may seriously compromise the effectiveness of clinical therapy and is another potential threat to public health.

To date, at least 69 optrA variants have been detected (Schwarz et al., 2021), and 7 OptrA variants were detected in this study. We found the RDK and DP variants were the common variants in *E. faecalis*. Previous studies suggested that the different OptrA variants might have an impact on the relative linezolid susceptibility/resistance of the respective isolates (Schwarz et al., 2021). A previous study demonstrated that enterococci strains (isolated from asymptomatic healthy humans) carrying the wild-type *optrA* gene or the RDK variant exhibited relatively high levels of resistance to linezolid compared to other variants (Cai et al., 2019). Moreover, another study demonstrated that strains with the RKD variant had linezolid MICs of 8–32 μg/ml, while those with the wild-type *optrA* gene showed MICs of 8–48 μg/ml (Wang et al., 2024). The RDK variant increased the MIC of linezolid (Li et al., 2020). We observed that isolates harboring the EDM and EYDNDM variants for linezolid MICs were 4 μg/ml, as observed in previous studies (Cai et al., 2015). The wild-type and other variants (RDK, DP, DD, EDM, and KLDP) were linezolid MICs of 8 μg/ml. Thus, distinct variants of the *optrA* gene may confer differential resistance to linezolid in enterococci.

In this study, the core structure *fexA*-*optrA* was found, which was also identified in various bacteria from humans, wastewater, and animals (Tang et al., 2021; Yang et al., 2024; Freitas et al., 2017; Abdullahi et al., 2023). Variations were distinguished by various flanking IS elements and other genes located between these elements. Mobile genetic elements, including IS1216E and ISVlu1, contribute significantly to the transmission of *optrA* (Partridge et al., 2018). *Tn554* mediation of *optrA* transfer has been identified (Kang et al., 2019). The *optrA* flanking structures were observed from different species, suggesting that the *optrA* cluster may jump via mobile elements, including transposon (*Tn554*), insertion sequences (IS1216E and ISVlu1), and transposase genes (*tnpY* and *tnpC*). The findings suggested that transposable elements, including ISVlu1, IS1216E, and *Tn554*, may be important in the transmission of *optrA* in the anorectal surgery ward.

Due to the limitations of second-generation short reads, we were unable to obtain and analyze the complete genetic environment including complete plasmids.

The intestine is a reservoir of drug-resistant genes. The detection of *optrA*-positive *E. faecium* from bile suggests that drug-resistant bacteria can also exist in the gallbladder upstream of the intestine (Deng et al., 2023). In the present study, the high carriage of *optrA*-positive *Enterococcus* in anorectal disease not only makes the treatment difficult but also may pose a potential human health risk. Hence, *optrA*-carrying enterococcal from the intestine needs further attention.

In conclusion, we report the high carriage rate of the *optrA* gene isolates from anorectal disease patients and present the genetic diversity. Different mobile genetic elements including *Tn554*, IS1216E, and ISVlu1 mediated the dissemination of *optrA*. The prevalence and spread of *optrA*-carrying enterococci among patients in the anorectal surgery ward should be actively monitored.

## Data Availability

Genome sequences of 15 Enterococci strains tested in this study have been deposited in the NCBI database under the BioProject accession number PRJNA1115951.

## Footnotes

^1^ https://cge.cbs.dtu.dk/services/all.php

^2^ https://cge.cbs.dtu.dk/services/PlasmidFinder/

^3^ https://software.broadinstitute.org/morpheus/

^4^ https://pubmlst.org

^5^ http://rast.nmpdr.org

^6^ http://blast.ncbi.nlm.nih.gov/blast

^7^ http://mjsull.github.io

^8^ https://github.com/marbl/parsnp

^9^ https://itol.embl.de
